# Registering Cosmetics? The Constitution of Legal Form and Injurious Substance in Canada (1945–1946)

**DOI:** 10.1177/09646639231173051

**Published:** 2023-05-15

**Authors:** Lara Tessaro

**Affiliations:** Kent Law School, 2240 University of Kent, UK

**Keywords:** Cosmetics, registration, material-temporal regime, injury, legal history, Canadian constitutional law

## Abstract

In midcentury Canada, legislative drafters, government lawyers, food and drug officials, and ministers grappled with cosmetics. Faced with constitutional concerns about cosmetic licensing, these actors drafted legislative amendments that would instead require cosmetics to be registered. In contrast to people or land, the registration of products, substances, or things has received little attention in sociolegal scholarship. Building on work investigating law's temporalities and materiality, this account traces how in-formed by the constitutional doctrine that apprehended substances through the legal form of prohibition, cosmetics were rendered in draft legislation as constituted of ingredients that may cause injury. Injury, in this account, is a material-temporal regime. Yet cosmetic injury was neither static nor singular, as it was catalysed differently by distinctive regulatory devices. This is shown by last-minute changes to the bill which retooled cosmetic registration, from an information extraction device for anticipating future harms, into a recording device for capturing latent harms.

In January 1946, Elmer Driedger, a lawyer with Canada's Department of Justice (DOJ), advised that the cosmetic licensing provision in the *Food and Drugs Act* was unconstitutional. Working closely with legal counsel and technical officers of the Department of National Health and Welfare (National Health), over the next months, Driedger drafted statutory amendments in an effort to render cosmetics as a constitutionally tractable object. This article traces the attempts of legislative drafters, government solicitors, food and drug officials, and a cabinet minister to formulate constitutional and statutory foundations for cosmetics regulation in Canada. How did federal officials in midcentury Canada grapple with cosmetics? Through what legal forms did they apprehend these substances? What regulatory devices would they contemplate using to govern cosmetics as legal things, and what material and temporal effects would be produced or signaled by these devices? In answering these questions, this story brings together the regulatory device of registration, Canadian constitutional doctrine, and injury as a material-temporal regime.

To protect the public from harmful cosmetics, Canadian officials would land, in 1946, on the regulatory device of registration. In critical legal studies, registration is often theorized as a technology of governmentality (Trabsky, 2022). Such theorization seems especially apt for registration processes that involve extracting data about the life course of individuals or their ‘personhood’ (Szreter and Brechenridge, 2012), such as birth and death registration (Smith, 2021), which data are aimed at demographic or population-level knowledge (Trabsky, 2022). Registration can target entities other than people, perhaps most obviously land (Holder and McGillivray, 2020; Keenan, 2017, 2019; Pottage, 1995). Moreover, products, substances, or things can also be registered, though this has received little attention in sociolegal or critical legal studies. In this legal-historical study, my inclination is to resist projecting governmentality onto scenes featuring Canadian health regulators in 1946, a location witnessing the explosion of the social welfare state and its institutions. My actors were constructing not only cosmetics law but the constitutional logics and legal practices underpinning those emerging institutions. In tracing legislation in the making, this story recognizes the historical contingency and institutional specificity of cosmetics and their regulation in Canada, concentrating on the events, practices, and discourses through which legal forms and injurious substances were being co-constituted.

Canada's constitutional division of powers does not expressly confer jurisdiction over ‘health’ either to Parliament or to the provincial legislatures. Therefore, to be constitutionally valid, health legislation must fall under some other head of federal power or provincial power enumerated in sections 91 or 92, respectively, of the *British North America Act, 1867* (*BNA Act, 1867*).^1^ The scope of these federal and provincial powers has of course expanded and contracted at different times in Canadian constitutional history, including as a result of judicial interpretation. In particular, the Judicial Committee of the Privy Council notoriously narrowed and neutered many Dominion powers over the first four decades of the twentieth century (Saywell, 2002). By the mid-1940s, federal power – especially, if not only, its power over trade and commerce – had been strangled by imperial judges. The Privy Council continued to act as a court of last resort for constitutional and civil matters during that decade (though criminal appeals to the Privy Council had been legislatively abolished in 1933). Thus, DOJ and National Health were wrestling with cosmetics at a moment when the federal social welfare state was rapidly expanding but its legislative authority had been consistently constrained.

This constitutional context would strongly channel federal officials’ efforts to construct a regulatory device capable of handling cosmetics and their hazards. The Privy Council had pronounced that the Dominion could not regulate a trade through licensing unless the licensing scheme was necessarily ancillary to the objects of a valid federal statute; licensing a business or trade was a matter of property and civil rights, which fell within provincial power (*Attorney-General for Canada v Attorney-General for Alberta,* 1916)*.* However, as will be elaborated, federal power over criminal law remained relatively robust, at least as of 1946, providing Parliament with authority to restrict the sale of harmful substances. Moreover, unlike licensing, registration had not been judicially impugned. As Parliament could enact criminal law in the face of hazards to public health or safety, federal officials were quick to apprehend cosmetics as injurious.

In describing how cosmetics registration would have formulated injury, my narrative is informed by historiographical and sociolegal engagements with law, time, and matter. As will be developed, this article conceives of ***injury*** as a material-temporal regime. Recent historiography on time and power expands on the concept of temporal regimes. As ‘orderings of time and its experience’, a temporal regime ‘refracts political, social, hierarchical, even aesthetic structures: it links temporal order, encodes power and politics, forges realities and limits of experience, contributes to the perpetuation of existing systems and the claims of new ones’ (Edelstein et al., 2020: 7). Temporal regimes are being detailed in imperial, colonial, and international legal history. Writing against the spatial metaphors and metrics of jurisdiction often privileged in international and imperial legal histories, Natasha Wheatley persuasively reveals temporal regimes in settler-colonial states that are multiple and conflicting, exploring how legal pluralism within purportedly singular states is marked by historical rather than spatial difference (Wheatley, 2020, 2021). Similarly seeking to redirect attention away from territoriality and towards temporality, Renisa Mawani nonetheless works to bridge the temporal with the spatial – if less so with the material – in colonial legal history (Mawani, 2019). She charts reconfigurations of time initiated by maritime navigation and consolidated by British colonial law through following the 1914 journey of the *Komagata Maru,* arguing that the uniform time of the British Empire was inaugurated through the juridical form of the ship. While these legal historians endeavour to hold time, space, and law together, the field has not engaged very deeply with matter. Tom Johnson has offered programmatic suggestions for how legal historians might embrace the material turn in the humanities and social sciences (2018). Building on insights from science and technology studies (STS) and legal geography, Johnson demonstrates how, in the context of the law of shipwreck in medieval Suffolk, ‘law projected a certain kind of materiality, and these projections manifested in material things’ (Johnson, 2015: 407; see also Tessaro, 2020). Outside of history, sociolegal scholarship has been approaching time and things together. In groundbreaking work, Emily Grabham conjoined STS, new materialism, and sociological and anthropological scholarship on time to examine how actors – both human and nonhuman – interrelate in legal assemblages so as to ‘brew’ legal times (Grabham, 2016). Other socio-legal scholars have also investigated connections between law, matter, and time, in pursuing material-temporal or spatial-temporal questions about legal practices (McNeilly, 2021; Sullivan, 2020; Valverde, 2015).

In tracing these historical events, this article also makes use of comparative methods. Registration is not compared across jurisdictions, however, but rather across other regulatory devices employed under the same statute, at the same time, by some of the same officials. Under the *Food and Drugs Act,* biological drugs were subject to licensing, while food and drugs were often required to conform to standards. Further, as will be seen, federal officials devised two distinct registration schemes. Comparison of these various devices draws out the diverse material and temporal effects that would be produced by cosmetic registration. In addition, comparing registration with licensing discloses a gendered element to how injury was being constructed by government lawyers. In using historical and comparative methods, I follow approaches to land registration that compare temporal effects manifested by registration (Keenan, 2017, 2019) and examine the transforming materiality of registration over time (Pottage, 1995).

My account draws on a range of primary sources. Notably, some of these sources have never been considered by other scholars or historians. In particular, the pith of this matter is Driedger's solicitor-client file, containing opinions, correspondence, and draft legislative text (Library and Archives Canada (LAC), RG13, Vol. 2635, file no. 9-150108 (Cosmetics File)). Public access to this file was only permitted in 2018; prior to that, the mid-century effort by federal departments to register cosmetics was kept secret.^2^ Other primary sources include material in the Department of Health fonds, Brooke Claxton fonds, Elmer A. Driedger fonds, and Privy Council fonds, all in the care of LAC; historical legislation; appellate decisions on the constitutional division of powers; contemporaneous journal articles; and statistical reporting on the Canadian cosmetics industry. Secondary sources on the business, cultural, and legal history of cosmetics, and on food and drug regulation in North America, flesh out my account.

The first part of this article describes and analyses the legal opinion given by Driedger in January 1946. He concluded that the existing provision authorizing licensing of cosmetics manufacturers was unconstitutional. Yet, as he explained, Parliament could nonetheless enact a statute prohibiting the sale of dangerous cosmetics, as *prohibition* was a legal form within Parliament's constitutional competence over criminal law. Despite accepting those cosmetics could be hazardous, Driedger's advice performed subtly gendered distinctions between drugs and cosmetics, validating licensing for the former but not the latter. The second part of this article narrates efforts by federal officials, over the winter and spring of 1946, to draft a bill that would bring cosmetics under the *Food and Drugs Act*. Central to this bill would be a prohibition of the sale of injurious cosmetics, as operationalized by the registration of cosmetic products.

## Licensing Legislation: Federal Officials Locate Constitutional Authority for Regulating Cosmetics

During World War 2, cosmetic sales exploded in the United States (Jones, 2010; McEuen, 2011; Peiss, 2011). Famously, the US War Production Board imposed restrictions on cosmetic production in 1942 only to rescind its order 4 months later, having ‘undoubtedly come to appreciate cosmetics … as vital to securing women's commitment to the war effort’ (Peiss, 2011: 244–245; Scott, 2006: 222). In Canada, the production of cosmetics steadily increased during the war. For companies engaged chiefly in manufacturing cosmetics and toilet goods, output leapt from $6,918,573 in 1939, to $18,992,908 in 1945, an increase of 275 percent (Canada, 1941; Canada, 1947). The industry's rapid growth coincided with women turning to ‘performances of femininity to demonstrate their commitment to the war effort’ (Tessaro, 2020: 5–6). Melissa McEuen has demonstrated how these patriotic performances produced and augmented racial hierarchies in wartime America, where a ‘genuinely “feminine” face was dictated by racial meanings and age’, and the ‘women considered most likely to possess or have the ability to create one were middle-class housewives’ (McEuen, 2011: 6). Once the post-war economic revival was underway, and as previously quotidian grooming habits morphed into stronger statements of national, gendered, and racialized identity, women's appetite for cosmetics rapidly grew (Jones, 2010: 202–203).

**Figure 1.**
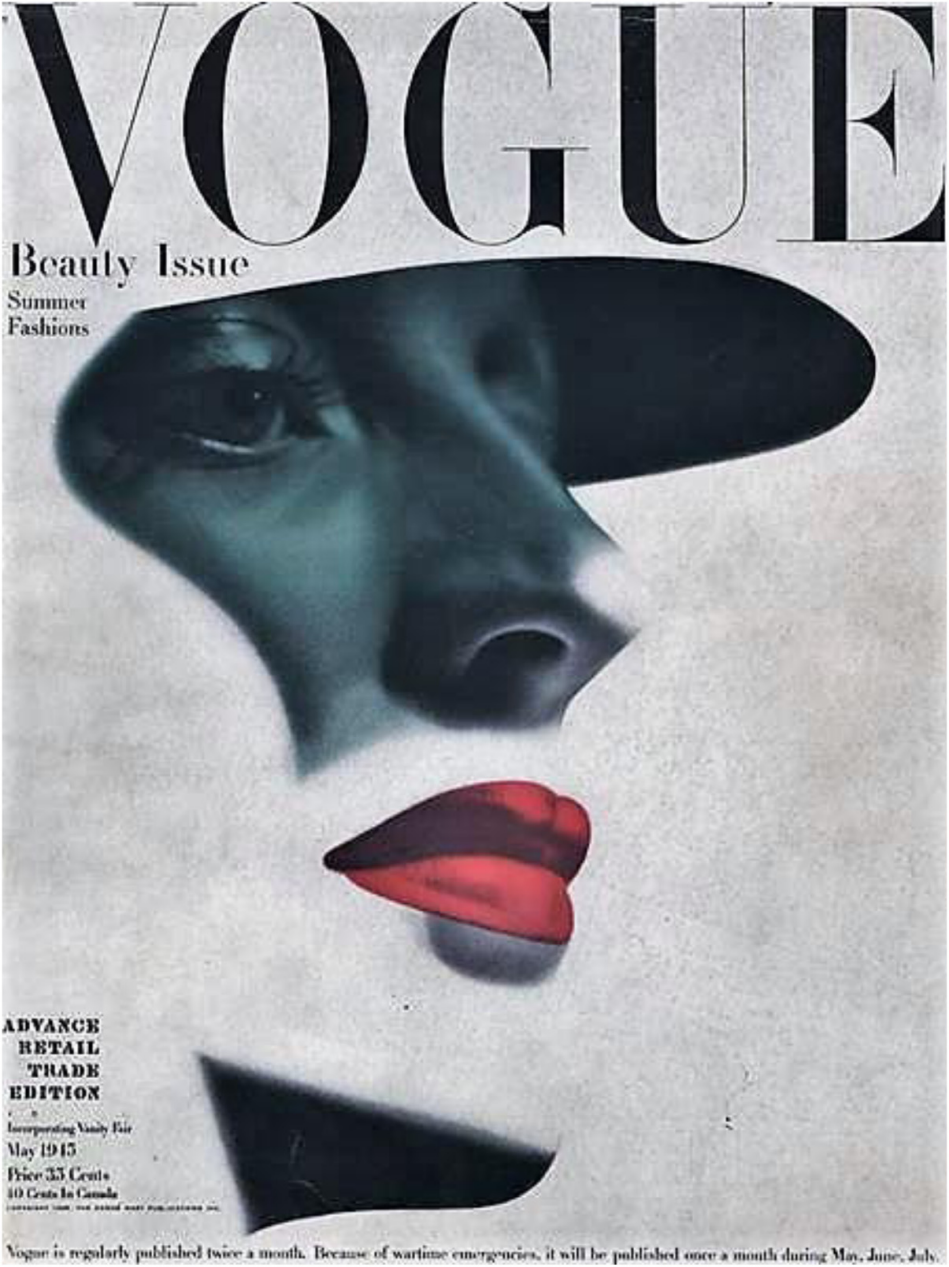
‘Vogue is regularly published twice a month. Because of wartime emergencies, it will be published once a month during May, June, July’. Cover: Vogue. 1945. *Vogue,* 105(9).

In 1945, no cosmetic trend was so indelible as the embrace by Western women of lipstick – preferably red and long-lasting (Ragas and Kozlowski, 1998: 24, 79). By one estimate, by 1948, 90 percent of women in the United States used lipstick (Peiss, 2011: 245). Red lipstick and patriotism were linked through images like that published on the cover of *Vogue* in May 1945, in which shading resembled the visor of a soldier's helmet (Figure 1). Though popular, lipstick was not harmless. It immediately attracted enforcement activity under the US Food, Drug, and Cosmetic Act of 1938. In 1939, the US Food and Drug Administration made high-profile seizures of lipstick, manufactured by the French firm Guerlain, containing the harmful ingredients of cadmium and selenium (Kay, 2005: 110–115). Cosmetic hazards were not limited to lipstick, as other products marketed exclusively to women were also proving dangerous. The Food and Drug Administration aggressively enforced its new law against eyebrow dyes, hair dyes, and mascaras with harmful ingredients. Moreover, in the early 1940s, physicians and medical researchers were documenting injuries associated with nail polishes, leg makeup, hair lacquers, and cold wave preparations (107–109; 115–122).

National Health was well aware of these hazards but, unlike the US, it had done little to address them. In 1939, statutory amendments had brought cosmetics under Canada's *Food and Drugs Act*, making cosmetics a class of drugs and empowering National Health to license cosmetic manufacturers (*An Act to Amend the Food and Drugs Act*, 1939). However, Canada had not subsequently proclaimed the cosmetics provisions into force (Proclamation, 1939).

In this milieu, National Health returned to cosmetics in the fall of 1945, when its Food and Drug Divisions drafted regulations governing the manufacture and sale of cosmetics (LAC, Chisholm to Varcoe, 22 January 1946, Cosmetics File; LAC, Driedger's 1945 Appointment Book, MG31-E39, Vol. 41). Among other things, these draft regulations prohibited the sale of cosmetics ‘containing antimony, arsenic, lead’ and, for certain products, they prohibited or restricted coal tar ingredients (LAC, Driedger to Varcoe, 25 January 1946, Cosmetics File).^3^ As a matter of form, these prohibitory regulations did not emulate British or American precedent. The US Food, Drug, and Cosmetic Act of 1938 prohibited the adulteration or misbranding of cosmetics, but it did not empower the prohibition of cosmetic ingredients through regulations. Such a power had been deleted from the US bill before it passed into law in 1938, ‘chiefly at the insistence of hair-dye producers (whose products contain ingredients often causing harmful reaction)’ (Cavers, 1939: 41).^4^ Britain, moreover, did not regulate cosmetics at all.

The Food and Drug Divisions consulted the cosmetics industry on the draft regulations in January 1946. In response, the Toilet Goods Manufacturers Association proffered a legal opinion by one of Canada's ‘most eminent constitutional lawyers’ (Saywell, 2002: 204; see also *New York Times*, 1946: 27). Aimé Geoffrion opined that the cosmetic licensing provision in the *Food and Drugs Act* was unconstitutional, as it did not fall within Parliament's legislative powers enumerated in s. 91 of the *BNA Act, 1867*, but rather within provincial jurisdiction. He sensibly argued that, without a constitutionally valid statute to empower them, the regulations could not be made (LAC, Chisholm to Varcoe, 22 January 1946, Cosmetics File).

On receiving the industry's opinion, Dr Brock Chisholm, the Deputy Minister of Health, promptly sought advice from the Deputy Minister of Justice, Frederick Percy Varcoe. In addition to seeking an opinion on the constitutional validity of the cosmetic licensing provision, Chisholm also requested advice on the validity of other statutory licensing schemes administered by National Health (LAC, Chisholm to Varcoe, 22 January 1946, Cosmetics File). These questions were assigned to Elmer Driedger. After joining the DOJ in the early 1940s, Driedger increasingly specialized in legislative drafting, receiving the newly created designation of Legislative Counsel in 1946. In future decades, Driedger would become Canada's most well-known practitioner and scholar of legislative drafting and statutory interpretation (MacLaughlan, 1995: 188–189).

In Driedger's opinion, the cosmetics licensing provision in the *Food and Drugs Act* was indeed unconstitutional. Doctrinally, ‘the licensing of manufacturing is a matter of property and civil rights’, which was a provincial matter, unless the licensing provision fell within some other enumerated head of power in s. 91 of the *BNA Act, 1867*. Driedger could find no such power. Under s. 91, the only potential heads of federal power were ‘trade and commerce, criminal law and the peace order and good government clause’ (LAC, Driedger to Varcoe, 25 January 1946, Cosmetics File). Of these grounds, his opinion unsurprisingly centred on the criminal law power in s. 91(27). In 1946, the criminal law power was not suffering the fate of other federal heads of power, such as the trade and commerce power, which had been decimated by numerous decisions of the Privy Council (and, to a lesser degree, of the Supreme Court of Canada). Driedger's advice centred on the Privy Council's decision in *Proprietary Articles Trade Association* v *Attorney General for Canada* (1931). Rejecting earlier judicial limits on the scope of criminal law power, *Proprietary Articles* had enlarged federal power to declare any act a crime – provided that the statute doing so, as a matter of form, contained both a prohibition and a penalty. ‘Criminal law connotes only the quality of such acts or omissions as are prohibited under appropriate penal provisions by authority of the state’, Lord Atkin had written, so that ‘[t]he criminal quality of an act cannot be discerned by intuition; nor can it be discovered by reference to any standard but one: Is the act prohibited with penal consequences?’ (1931: 90). Despite the breadth of the criminal law power as expanded by Lord Atkin, the licensing section could not be sustained. As put by Driedger, ‘nothing is prohibited’. The constitutional difficulty was that ‘Parliament has not said enough’ with respect to cosmetics. Nevertheless, the drafter was confident that Parliament could validly enact a future statute prohibiting the sale of dangerous cosmetics (LAC, Driedger to Varcoe, 25 January 1946, Cosmetics File).

Doctrinally, Driedger's conclusion was uncontroversial. Nonetheless, when the opinion is analysed through a lens of gender rather than doctrine, an internal tension is revealed. On the one hand, Driedger assumed that cosmetics could be sufficiently harmful so as to justify intervention under the Dominion's criminal law power; on the other, his opinion constructed cosmetics as *not* injurious through an implicit comparison with drugs. Recall that National Health had also sought advice on the constitutionality of other statutory licensing schemes. This Driedger provided within the same opinion letter. In advising that *The Proprietary and Patent Medicine Act* and the *Opium and Narcotic Drug Act* were both constitutional, he relied on the fact that courts had upheld the validity of these acts, in decisions classifying these statutes as criminal law.^5^ More striking was his analysis of the licensing of biological drugs, which had not been judicially considered. As with cosmetics, licensing of biological drugs was provided for by the *Food and Drugs Act* (RSC 1927, ss 6(3) and (4))*.* In Driedger's view, there was ‘no difficulty’ with this scheme. Licensing such drugs was ‘probably very necessary’ as ‘these products are no doubt injurious to health unless they are carefully manufactured, tested and labelled under proper conditions’. As such, the licensing provisions were ‘merely ancillary to the main purpose of the Act and are designed to protect the health and life of the public’ (LAC, Driedger to Varcoe, 25 January 1946, Cosmetics File). Finally, he noted that the BC Court of Appeal had upheld the validity of the *Act* in *Standard Sausage v Lee* (1933, 1934) (although that decision offered little real assistance as the statute's licensing provisions were not at issue.)

In what respect, one might ask, did these considerations not apply equally to cosmetics? After all, National Health regulators and scientists viewed some cosmetics as injurious to health, and they believed that licensing of cosmetic manufacturers was necessary to protect public health – or to be more precise, they believed this was necessary to protect the health of women. The harmful products that had created concern over the previous decade – the lipsticks, mascaras, eyebrow and hair dyes, and cold waves – were marketed to women, and the injuries recorded in medical journals and recounted in congressional hearings had been suffered by women. Likewise, the chemicals targeted by National Health's draft regulations were found in products marketed exclusively to women; antimony was used in eye make-up, arsenic and lead in face powder, and coal tar dyes and heavy metals in eyelash, eyebrow, and hair dyes (Kay, 2005: 61–62, Jones, 2010, 63). Working to achieve his client's objectives, Driedger would have envisioned cosmetic users – or at least the users of *dangerous* cosmetics – as primarily female. Thus, in essence, his opinion insisted that the licensing of harmful women's products must be held to strict standards of constitutional justification.

By contrast, for biological drugs, Driedger was willing to automatically deem licensing to be necessary, without knowing whether these drugs were harmful and without even considering whether licensing was ancillary to any underlying criminal prohibition. Put simply, biological drugs, presumed to be used by anybody, were held to a lower constitutional standard in his legal opinion than were women's products. By deploying two different constitutional standards for the two product classes, Driedger impliedly constructed cosmetics and biological drugs as constitutionally different. Moreover, this obverse reasoning used for cosmetics as compared to drugs is closely juxtaposed within the same memorandum. In this way, when read as a whole, the opinion positions cosmetics and drugs relationally, constructing them as a near-antonymous dyad. This juxtaposition served to code the concept of ‘drug’, within the opinion, to mean ‘not cosmetics’. Drugs signified products used by everyone and presumed to be dangerous. Cosmetics, in turn, meant ‘not drugs’, signifying products used primarily by women and presumed to be safe in comparison. These binate, relational meanings, arising from the structure of the opinion and from Driedger's willingness simply to deem licensing necessary for biological drugs, were in implicit tension with the lawyer's own opinion that Parliament had jurisdiction to prohibit the sale of dangerous cosmetics under its criminal law power – as that opinion, of course, was premised on cosmetics being actually or potentially harmful.

From this first sustained encounter between cosmetics and Canadian constitutional law, the nature of cosmetics as injurious substances was already a slippery matter. If Canada was going to regulate cosmetics constitutionally, then injury would need to be pinned down. Over the coming months, DOJ and National Health would work to bring cosmetics within federal jurisdiction. In contrast to the implicit distinction within Driedger's legal opinion, National Health would quickly analogize cosmetics to drugs; moreover, to avoid the constitutionally controversial device of licensing, National Health would propose that cosmetics be subject to registration.

## Formulating Cosmetics: Registration, Prohibition, and Regimes of Injury

With licensing called into constitutional question, how should cosmetics be regulated in Canada? In the winter and spring of 1946, Canadian legislative drafters, government solicitors, and food and drugs officers would wrestle with this question. Driedger had established that whatever regulatory mechanism was devised to handle cosmetics would need to be integrated within a constitutionally valid statutory scheme, and any such scheme would need to be designed around a criminal prohibition rendering cosmetics as injurious. Therefore, developing and drafting amendments to the *Food and Drugs Act* would necessitate carefully assembling relations between constitutional authority, the legal form of prohibition, and the regulatory device of choice.

The device chosen was registration. In this second part of the article, I trace how cosmetics registration was conceived, how registration would interact with prohibition, and how registration and prohibition morphed as a result of ministerial involvement, exploring throughout how these regulatory arrangements would re-order matter and time. In the first section below, federal officials strike upon the idea of requiring cosmetics to be registered prior to sale. As registering cosmetics was largely without precedent, officials grappled with how registration might work in the course of developing a draft bill over February and March 1946. The second section looks at injury as a material-temporal regime. Injury was the linchpin to Driedger's cosmetics bill. The prohibition of injurious cosmetics, when combined with the registration scheme, would catalyse a new regime of injury, one that moved away from the materiality and temporalities cemented by the existing devices of standards and labels, which had been long employed to regulate food and drugs in Canada. As conceived in the bill, the prohibition and registration scheme would together foster a material-temporal regime of *anticipation,* in which injury was always on the verge of materializing. In May 1946, the Minister of National Health and Welfare requested last-minute changes to the bill. The final section explores how those changes would have re-tooled registration so as to capture already latent hazards, thus reformulating injury, materially and temporally, as *latency.*

### Registration as a Device for Extracting Information from Industry: The First Draft of the Cosmetics Bill (February-March 1946)

Driedger's opinion did not diminish National Health's desire to regulate. On the contrary, the Food and Drug Divisions was impatient for statutory authority, in whatever form, that would allow it to impose controls on cosmetics. This section traces how National Health took up DOJ's offer to draft amendments to the *Food and Drugs Act.* In particular, National Health proposed a new device, one uncontaminated by appellate jurisprudence on the constitutional division of powers*,* for regulating cosmetics. In lieu of licensing, the Department proposed that cosmetics instead be registered (LAC, Chisholm to Varcoe, 19 February 1946, Cosmetics File).

In one way, this proposal was unprecedented, reflecting an experimental effort to come to grips with constitutional constraints. In the 1940s, only a few countries regulated cosmetics in any fashion. The United States did, but it did not require registration of cosmetics. Indeed, it appears that only Peru required the registration of toilet preparations (LAC, Gilchrist to Teevens, 6 March 1942 (sic) with decree, and Gilchrist to Teevans, 22 October 1942, with decree, RG29, Vol. 245, file no. 336-4-1). At a sub-national level, by 1946, at least two US states, Maine and Louisiana, required some cosmetics to be registered (Mock, 1946: 64). However, National Health did not develop its proposal to register cosmetics based on approaches taken by Peru, Maine, or Louisiana. Moreover, neither food nor drugs were typically registered in other Western jurisdictions.

Rather, the precedent for this proposal was another statute administered by National Health. First enacted in 1908, *The Proprietary or Patent Medicine Act* governed proprietary medicines. These nostrums and tonics, marketed for relief of minor illnesses and ailments, were also known as ‘secret formula’ medicines. Despite that it contained a licensing power, *The Proprietary or Patent Medicines Act* had been upheld as constitutional by the Ontario Supreme Court in 1917, its licensing requirements aimed at the protection of the public rather than at regulating the trade (*Rex v Warne Drug Co. Ltd, 1917*: 789). Central to this act was a requirement that proprietary medicines be registered with National Health, with the ingredients of such medicines disclosed by the manufacturer in its registration application. Once registered, the manufacturer could receive an annual license allowing the product's sale in Canada (Soucy, 1953: 707–708, 713).^6^ In 1919, the registration requirements had been expanded to apply to preparations intended not only for internal but also external use (Soucy, 1953: 706, 709). National Health officials would have viewed the mandatory registration of cosmetics as an incremental step from the registration of ‘secret formula’ ointments and liniments. Dr Chisholm articulated the scope and purpose of the Department's registration proposal as follows:

Such registration provision would by itself, or by regulations require the qualitative formula of the ingredients as a condition of registration. It would not be sufficient merely to require dangerous cosmetics to be registered. It is impractical to devise an exhaustive list of *what ingredients may be actually, or potentially, dangerous* and it is not considered sufficient that this choice be left to the manufacturers. Accordingly…each article of cosmetics should be registered with the Department and a list of the ingredients shown in the registration application. (Chisholm to Varcoe, 19 February 1946, Cosmetics File, emphasis added).

Just as with licensing, what National Health most desired from registration was to extract information from cosmetic manufacturers. Food and drug officers wanted to know which cosmetics were being put on the market, what ingredients those products contained, and if those ingredients were hazardous, as without this information, the Department's ability to monitor or control cosmetics would be hamstrung. To make the proposal appear less radical, Chisholm argued that registration would transfer to industry the duty to report information about the composition of cosmetics that, if pressed, the Department's technical officials could always gather themselves by conducting ‘exhaustive analysis of the articles sold’. This argument was slightly disingenuous. With only one technical officer dedicated to cosmetics research in the Department, and faced with rapid technological innovation by the industry, such analysis may have been hypothetically possible but was practically unrealistic, at least on a scale meaningful to enforcement. Besides, National Health was not chasing ingredient disclosure merely for knowledge's own sake. Rather, as its deputy minister explained, knowing the composition of cosmetics would enable officials to make a ‘ready decision’ to restrict the sale of products that ‘might or might not prove to be injurious’ (LAC, Chisholm to Varcoe, 19 February 1946, Cosmetics File).

DOJ agreed with National Health's registration proposal. In March 1946, Driedger worked with food and drug officials to ready a bill to amend the *Food and Drugs Act*.^7^ National Health's lead was Robert Curran, the Department's new in-house legal adviser, supported by technical officers in the Food and Drug Divisions (Curran, 1953: 5). Despite the novelty of the registration proposal, discussions between legal and technical officials rapidly turned towards drafting solutions that would be quick and easy. Food and drug officers were comfortable with the *Act*'s elaborate provisions governing procurement and analysis of samples, investigations, and enforcement, and they wanted to squeeze cosmetics to fit within those familiar provisions, particularly those applicable to drugs. Senior officials favoured this approach for its political pragmatism, as Chisholm wanted amendments that he could give to his minister expeditiously. However, amending and re-enacting dozens of existing provisions to apply each of them to cosmetics could appear, on its face, to be ‘a formidable amendment to the Act’, which was not something that Brooke Claxton, the Minister of National Health and Welfare, wished to be seen as sponsoring. Thus, beyond the registration requirement, Claxton's officials preferred a *mutatis mutandis* provision that would extend existing provisions to cosmetics (LAC, Curran to Driedger, 1 March 1946, and Driedger to Varcoe, 19 March 1946, Cosmetics File).

National Health's preferred approach also reflected that, as in 1939 when cosmetics had been legislated as a class of drugs, food and drug officials continued to see many continuities between drugs and cosmetics. Prior to the therapeutic revolution, beauty and health ‘had been closely integrated’ (Jones, 2010: 243). In the early 1940s, drugs and cosmetics were steadily being manufactured in factories rather than compounded in pharmacies, with these industrial facilities heavily clustered in Montréal and Toronto. Like drugs, many mass-market, non-luxury cosmetics were being retailed in drugstores and, increasingly, in supermarkets (Jones, 2010: 116, 211; Peiss, 2011: 245). Like drugs, cosmetics could contain dangerous ingredients; like drugs, a growing technical capacity existed to identify a product's ingredients and to test their toxicity (Kay, 2005: 109–123). Some large multinational companies produced both cosmetics and drugs. In one striking example of this crossover, Swiss pharmaceutical giant Hoffman-La Roche ‘launched Pantene shampoo as a by-product of the synthesis of the vitamin panthenol in 1945’ (Jones, 2010: 244).

These institutional preferences and industrial continuities were once again leading National Health down a path towards unconstitutional legislation. When asked by DOJ to identify the specific sections of the *Food and Drugs Act* that should be extended to cosmetics, food and drug officials identified nearly all existing provisions that applied to drugs (LAC, Curran to Driedger, 1 March 1946, Cosmetics File). Yet these officers also suggested that the two main statutory prohibitions applicable to drugs – namely, adulteration and misbranding – did not need to be extended to cosmetics. If adopted, this suggestion would leave cosmetics without any applicable prohibition; if nothing was prohibited, then the extension of the *Food and Drugs Act* to cosmetics could not be constitutionally justified under the federal criminal law power.

Driedger was determined to craft amendments that would set a constitutional foundation for federal authority over cosmetics. On March 19, 1946, he finished his first full draft of the bill. In addition to extending some existing provisions as National Health wanted, Driedger also created a new, standalone part specific to cosmetics. To ensure constitutionality, at the core of this part was a prohibition against the sale of injurious cosmetics. Ancillary to this prohibition was the mandatory registration of all cosmetics. Manufacturers would need to register their cosmetics with National Health prior to sale and, critically, registration would require cosmetic firms to disclose information about the ingredients in those products. Functioning as a primer to the bill's foundation, registration would enhance and actualize the prohibition of injurious cosmetics – in short, registration would make the prohibition stick*.* On top of prohibition and registration were layered a rich palette of regulatory powers, which would empower the federal government to regulate cosmetic packaging and labelling, to prescribe standards, and to restrict the use of ingredients that may be injurious. Together, these powers aimed to provide full regulatory coverage (LAC, Driedger to Varcoe, 19 March 1946, Cosmetics File). In this way, Driedger's bill assembled relations between constitutional authority, the legal form of prohibition, and the device of registration. ‘In-formed’ by constitutional doctrine (Barry, 2005; Delaney, 2021), cosmetics were being composed as a mixture of knowable ingredients with the capacity to cause injury and that could therefore be registered and, if need be, prohibited.

### Mobilizing Injury as a Material-Temporal Regime: Federal Officials Struggle with the Meaning and Consequences of ‘Injurious’ (March-April 1946)

Even as Driedger finished his first full draft of the bill, on March 19, 1946, he was still struggling to pin down the meaning of ‘injurious’. To be constitutionally valid, the amendments had to render cosmetics as potentially injurious substances. Conceptually, injury was critical – to fasten together constitutional authority, prohibition, and registration, injury would be the linchpin. In the section that follows, injury will be explored through the drafting efforts of federal officials in the spring of 1946. DOJ and National Health debated how and where the bill's provisions should characterize cosmetics as injurious. Their debate opens a window into how different regulatory devices, employed under the *Food and Drugs Act,* enacted varied material and temporal effects. Standards and labels, traditionally used to govern both food and drugs, materialized the past, while the regime of injury emerging for cosmetics brewed *anticipation* of imminent harm.

An injurious substance is readily grasped as material, and an injury is easily comprehended as physical or bodily. However, an injury also mobilizes time. ‘When did the injury occur’ is a question just as cognizable as ‘what was the injury’ or ‘where was the injury’. Furthermore, asking this question promptly invokes the multiplicity of injury's temporalities, as common articulations of injury are frequently ‘distinguished with reference to time’ (Morris, 2008: 399). That is, injury can be experienced as acute – arising suddenly, spontaneously, or swiftly, perhaps with a short duration – or experienced as ‘chronic’ – emerging through repetition, recurrent, and characterized by longevity or even lifelong duration (Jowsey, 2016). In simultaneously ordering experiences of temporality and corporeality, injury is a material-temporal regime. Moreover, as will be explored through this section and the next one, injury could manifest as multiple material-temporal regimes, even within the same doctrinal or statutory setting, as diverse regulatory devices like registration, standards, and labelling reordered matter and time.

For Driedger, the constitutional necessity and centrality of the prohibition against the sale of injurious cosmetics meant that the word ‘injurious’ demanded a statutory definition. In memoranda in his file, he casually equated injurious to ‘dangerous’, ‘hazardous’, or ‘harmful’. In drafts of the actual bill, the definition of injurious was in continual flux. His first draft, in March, assigned the word ‘injurious’ three distinct meanings. Two of the three meanings were stated in a two-part definition section: First, injurious would mean ‘declared by regulation to be injurious’; second, it would also mean ‘not prepared, packaged or labelled in accordance with the regulations’. For this second part of the definition, the rationale was that foods or drugs labelled contrary to regulations were deemed to be adulterated in the existing *Act*, and it was felt ‘necessary to incorporate the same idea with reference to cosmetics’. For reasons discussed, food and drug officials remained keen to slot cosmetics into the existing framework, without much if any consideration of how the concept of adulteration might differ from that of injury. However, for the first part of the two-part definition, Driedger changed his mind at the last minute. Right before sending his first full draft to his deputy minister on March 19, he took a pen to the typewritten draft and changed the first part to simply read ‘injurious to health’ (LAC, Driedger to Varcoe, 19 March 1946, Cosmetics File).

Apart from bundling labelling rules into this definition of injurious, it should be noted that Driedger's bill did *not* make labelling a key regulatory device. There was no effort to create any duty to disclose the ingredients of cosmetics on labels, which was unsurprising, as there was no discourse around consumers having a ‘right to know’ the constituents of products in the 1940s. The only people entitled, by this bill, to know the ingredients in a cosmetic would be National Health officials. When first proposing the registration scheme to DOJ, National Health had bolstered its pitch for requiring the industry to disclose ingredients by emphasizing that ‘this information would be confidential in the Department’ (LAC, Chisholm to Varcoe, 19 February 1946, Cosmetics File). Labels would have made public what registration would keep secret.

Finally, in addition to the two-part definition, Driedger provided a third articulation of injurious. In a separate prohibition, he provided that ‘every cosmetic shall be deemed to be injurious if its quality falls below the standard or its ingredients are present in a quantity not within the limits of variability fixed by the Governor in Council’ (LAC, Driedger to Varcoe, 5 April 1946, Cosmetics File). Again, this analogized ‘injurious’ to ‘adulterated’ in adapting the existing framework for food and drugs which, among other things, deemed a food adulterated if its ‘strength or purity’ fell below a prescribed standard, and deemed a drug adulterated if it differed in ‘quality or potency’ from a regulated standard (*Food and Drugs Act,* 1927, ss 4(g) and 6(4)). In this way, the first full draft of the cosmetics bill adopted traditional approaches to standards and labels, using them as yardsticks for deeming a cosmetic injurious (rather than adulterated).

On March 26, 1946, Driedger's first full draft of the bill was submitted to National Health (LAC, Varcoe to Chisholm, 26 March 1946, Cosmetics File). In the days that followed, National Health pushed for a few revisions. Most noteworthy was its requested revision to the prohibition of the sale of injurious cosmetics. The Department asked that the prohibition be expanded to indicate – as the above-mentioned provisions already did – that a cosmetic would be deemed injurious if its quality fell below a prescribed standard or if it was not labelled in accordance with regulations. As National Health acknowledged, its proposed addition was wholly duplicative. Nonetheless, its deputy minister argued that duplicating these words would assist laymen in administering the *Act* (LAC, Chisholm to Varcoe, 2 April 1946, Cosmetics File). Driedger viewed the addition as ‘unnecessary and superfluous’, and the DOJ opposed it on that basis (LAC, Driedger to Varcoe, 5 April 1946 and Varcoe to Chisholm, 5 April 1946, Cosmetics File).

As may be apparent, National Health wanted to duplicate only *half* of the two-part definition of injurious. Curiously, it did *not* ask the DOJ to repeat the phrase ‘injurious to health’. Chisholm offered no explanation for why his officials thought the prohibition section should repeat references to standards and labels yet need not replicate that more novel phrase. This moment, though minute, gives a glimpse into the material-temporal regime of injury emerging for cosmetics, and how this regime diverged from older material-temporal orderings enacted through standards and labels. The Department's food and drug officers were long accustomed to enforcing adulteration and misbranding, the two offences at the heart of the existing statutory regime, and were very familiar with standards and labels. Put simply, adulteration often policed whether a food or drug was made in accordance with a standard set down in either pharmacopoeia or regulations, while misbranding commonly policed whether a food or drug was falsely or deceptively labelled or packaged so as to appear other than its true character. Offences were committed when a product deceitfully deviated from an established recipe codified by a standard, or when a product misleadingly masqueraded, through its label or package, as something other than what it truly was. When food and drug officials regulated standards – akin to ‘time-honored recipes whose authority rested on familiarity and tradition’ – they performed lawful food and drugs as ‘pure’ or ‘authentic’ substances that were free from ‘chemical corruption of that authentic purity’ (Frohlich, 2021: 306, 307). Lawful substances conformed, in their material and moral essence, with what those substances were meant to be (Frohlich, 2021: 307; Rees, 2021: 91, 107). Standards and labels measured a food or drug against whether it corresponded to the purity and authenticity of the past, as baked into a standard and truthfully represented on a label. In so doing, these devices stabilized a material-temporal regime that was fixed, standardized, and looked backwards to yesteryear.

By contrast, a prohibition against the sale of cosmetics containing ingredients that were injurious to health was largely indifferent to the authenticity or purity of a registered substance. There were no standard ‘recipes’ for cosmetics, handed down over generations, against which their safety could be measured; to the contrary, cosmetic formulations, especially for perfume and other luxury items, were often closely guarded innovations. Prohibition, as operationalized by the device of registration, was instead concerned with the substance's anticipated material-temporal effects in the world to come – rather than asking what a cosmetic ***was****,* this device asked what a cosmetic ***would do***. What would ‘come to matter’, under this bill, was not a chemical's present being but its future doings (Barad, 2003). Would the ingredients in this cosmetic, when released from the package, cause injury? When the mixture mingled with a woman's body, would it cause her to lose her hair, develop a rash, go blind? Such questions speculated about the near future. Such questions could not be answered by interrogating if a product was what it was *meant to be* or what it *claimed to be*, even though such interrogations were conventional when administering and enforcing the *Act* for food and for drugs.

The prohibition of injurious cosmetics, when combined with the registration scheme, would produce a different material-temporal order. For these more mysterious, novel, slippery substances, whose worldly capacities were yet to be revealed, the scheme of the bill would brew another temporality, one of *anticipation*. With anticipation, ‘the not-yet-future reorients the present’ (Murphy, 2013; see also Adams et al., 2009). In contrast to traditional regulation of pure food or standardized drugs, the bill's registration scheme, when conjoined with the prohibition of injurious cosmetics, would slide the future into how cosmetics were apprehended in the present. The cosmetics bill gazed beyond dangers known in the present and toward harms anticipated to materialize imminently. In contrast to older ways of regulating, how the bill mobilized injury forecast the arrival, in post-war decades, of the risk regulation that so often typifies health law and environmental law in the twenty-first century (Stokes, 2021).

In drawing together constitutional authority, the legal form of prohibition, and registration of all cosmetic products, the bill mobilized a material-temporal regime in which injury was imminent and anticipated. Whether a cosmetic was safe was a matter of what its ingredients would shortly get up to. As will be seen in the next section, however, as political and epistemic contestations over registration unfolded later that spring, these legislative arrangements would come undone.

### Registration as a Device for Recording Latent Hazards: the Minister Directs Changes to the Bill (May–July 1946)

By the middle of May 1946, the bill had been finalized, approved by DOJ and by National Health, and sent off for printing (LAC, Curran to MacNeill, 25 May 1946, Cosmetics File). All that remained was for Claxton to secure the Cabinet's agreement, at its meeting on May 22, for the bill to proceed to Parliament. However, following that Cabinet meeting, Claxton directed changes to the bill. As explored below, these changes would have morphed cosmetics registration from a device for extracting unknown information from industry, into a ledger to record already known hazards. As the tool of registration morphed, so would its material and temporal effects. In the process, prohibition and injury – the legal form and the material-temporal regime – would become even more tightly tethered in Driedger's drafting practices.

Elected in 1940 to represent a riding in downtown Montréal, Claxton was appointed to Cabinet four years later as the Minister of National Health and Welfare. He had previously practiced commercial law, and, until his Cabinet appointment, had also been a law professor at McGill University. In the elite political and intellectual circles in which he travelled, Canadian nationalism and public welfare reforms were related topics of fevered discussion (Bercuson, 1993). Throughout the 1930s, Anglo-Canadian legal elites were increasingly incensed by what they perceived as Britain's ongoing legal imperialism, its influence maintained through the Privy Council. They fumed as the Privy Council repeatedly struck down, on division of powers grounds, important public welfare statutes enacted by the Dominion Parliament in the wake of the Depression (Scott, 1937). Claxton's longstanding interests in the law of federalism – advanced in law reviews, a report for the Royal Commission on Dominion-Provincial Relations, and, on occasion, appellate courtrooms – were never just scholarly or professional but always political (Claxton, 1932 Gouin and Claxton, 1939; *Re Regulation and Control of Radio Communications,* 1932). A member of the ‘government generation’ (Owram, 1986), he advocated social and economic reform through the state yet was forced to watch as the federal government's powers were neutralized by foreign judges (Bercuson, 1993; Claxton, 1935).

To the surprise of his officials, upon his return from the Cabinet meeting, the social reformer balked on certain policies propounded by the bill. At that meeting, ministers had discussed manifold matters, from Canadian attendance at US atomic bomb tests, to revoking orders made under emergency wartime powers, to the king's birthday (LAC, RG2, Series A-5-a, Vol. 2638, Items 31876, 6901, 6907 and 6896). Meeting records leave it unclear whether the cosmetics bill was actually debated, but it may not have been; apart from a bill to amend the Small Loans Act, the other five bills meant to be considered seem to have received, at best, very short shrift (LAC, R165, RG2, Vol. 65, file no. C-20-5, CD no. 209; LAC, RG2, Series A-5-a, Vol. 2638, Item 6899). Whatever happened, Claxton came out of the meeting determined to scale back the bill.

The minister was especially troubled by the registration scheme. After the Cabinet meeting, he worried that ‘registration of all cosmetics, irrespective of their ingredients, may prove contentious’ (LAC, Curran to Varcoe, 27 May 1946, Cosmetics File). With whom did he predict contention? The archives disclose no evidence of lobbying, that spring, by the Toilet Goods Manufacturers Association or its members. That said, objections from the industry, once it became aware of the bill, were easily anticipated. Equally foreseeable was contention in Cabinet. From the outset, Claxton had resisted advancing a bill that his colleagues might view as too substantial. Though he had helped to shepherd the *Family Allowances Act* through Parliament as a parliamentary assistant (Whitton, 1944: 416), he had not tabled a bill as a minister. Thin-skinned and sensitive to criticism, Claxton was still bruised by his Department's failure to establish a national health insurance program the year before (Bercuson, 1993; MacDougall, 2009); furthermore, just a few weeks earlier, the Cabinet had decided that his department's other public health initiatives, like its bill on old age pensions, should be ‘dropped for the present’ (LAC, RG2, Series A-5-a, Vol. 2638, Item 6872.) Claxton would have been anxious, in this moment, to avoid controversy.

However, the policy changes that Claxton then requested to the registration scheme reveal a superficial engagement with the bill's structure and internal logic. Likely not realizing the full implications of this instruction, he told his officials to modify the bill so that it would only require registration of cosmetics containing ingredients ‘which may be harmful’. Relatedly, he directed that a schedule of harmful ingredients is created by regulation, and that the Governor in the council be empowered to add ingredients. These modifications would make the bill less prohibitory and more permissive as, notably, they would allow products containing harmful ingredients to be registered without any restrictions on their sale to the public (LAC, Curran to Varcoe, 27 May 1946, Cosmetics File).

In Driedger's draft bill, registration had originally been devised as an epistemic tool that would enable National Health to gather information on the constituents of all cosmetics offered for sale in Canada. That original version was premised on the fact that the industry held information that government regulators lacked, such that registration would be an extraction device, meant to squeeze out information from manufacturers. The minister's intervention retooled registration, subtly yet significantly, by shifting the site of knowledge from industry to government. The changes that Claxton directed presumed that National Health would *already* possess information about harmful ingredients *before* a cosmetic was ever registered. In his version, rather than registration acting as a precondition to knowledge of potentially harmful ingredients, the temporal relation was reversed: existing knowledge of harmful ingredients would be a precondition to registration. As reconceived by the minister, registration would serve as a ledger, ensuring that products containing known chemical hazards were recorded.

The temporality forecast by this second registration scheme aligns with what Murphy calls *latency*. As Murphy puts it: ‘To be latent is to be “not yet:” a potential not yet manifest, a past not yet felt’. Latency ‘names the wait for the effects of the past to arrive in the present’ and as such is ‘the inverse temporal orientation of anticipation’ (2013). Latency is not only temporal. No longer anticipating possibilities of future injury, cosmetics registered under this revised registration scheme would materially brim with latent harm. Such cosmetics would now be the material residue of past industrial production, their harmful constituents crystallized in a schedule (Boudia et al., 2022). Reformatted in this way, registration would involve National Health documenting known chemical hazards, latent and lurking in cosmetics circulating through the market, recording the past as its residues seeped into bodies in the present.

As Claxton wanted to take the modified bill to Cabinet as soon as possible, Driedger promptly revised it, assisted by Curran, the National Health solicitor. As instructed, they drafted registration provisions that empowered a schedule of injurious substances and required cosmetics containing those substances to be registered. However, the two solicitors quickly realized that, as a consequence of the minister's instructions, an internal inconsistency would arise within the bill. On the one hand, under the new registration provisions, manufacturers would be allowed to sell cosmetics containing ingredients that may be ‘injurious to health’ (provided, of course, that they first registered those products). On the other hand, under the central prohibition section, manufacturers would be prohibited from selling cosmetics containing any ingredients ‘injurious to health’ (LAC, Curran to Varcoe, 27 May 1946 and Varcoe to Chisholm, 5 June 1946, Cosmetics File). Brooke Claxton, for all his legal and constitutional savvy, had introduced a fundamental flaw into Elmer Driedger's carefully drafted scheme.

To escape this conundrum, Driedger recommended one final revision, on June 5, to salvage the bill's scheme. Without mentioning this internal inconsistency, he advised that the word ‘prohibited’ be substituted for the word ‘injurious’ in a number of provisions. Driedger was quietly doubling down on his prohibition. His substitution meant the bill would prohibit the sale of cosmetics containing ‘prohibited ingredients’ instead of injurious ingredients. This allowed him to confirm that the bill was constitutional, because, as a matter of form and consistent with *Proprietary Articles,* the bill continued to contain a prohibition plus a penalty for its breach.

In substituting ‘prohibited’ for ‘injurious’, Driedger's final revisions clearly reproduced that doctrine. Beyond that, though, his substitution reveals the ontological and material power of legislative drafting. After months of repeatedly drawing prohibition and injury together, as an ever-more-tightly paired coupling in opinions and draft legislation, legal form and injurious substance had now explicitly converged. Examining how technical legal practices can perform metaphorical and material realities as if these were separate, Annelise Riles has analysed conflicts of laws doctrine as an example of how, following the work of anthropologists Roy Wagner and Marilyn Strathern, an ‘internal transformation of symbolic form produces, as its symbolic effect, an “actual” tool out of a metaphorical one’. Wagner had shown that when ‘used in new ways, symbols are differentiated from their “context”’, in the process becoming individuated as a ‘thing’ that is perceived as a standalone and material object (Riles, 2005: 1022). Similarly, Strathern had analysed how this ‘literalization of previously metaphorical conceptual relations’ was a central modernist move of ‘making explicit’ what had been implicit knowledge practices (Riles, 2005: 1023; see also Grabham, 2022: 5–6).

Driedger's drafting practices lay bare these theoretical insights, especially in this final transformation of the previously metaphorical relations between prohibition and injury in the bill. His inclusion of a prohibition in the bill had stood in as a metaphor for federal constitutional authority, as prohibitions symbolized the validity of any federal legislation in which they were found. Various prohibitions had long been embedded within the *Food and Drugs Act,* signaling that the targeting activity was harmful and that the Dominion had constitutional authority to legislate. These prohibitions were not, themselves, the regulatory tool; rather, their presence in the statute implicitly validated the more tangible, operative regulatory devices like labels and standards through which food and drugs were handled. However, to preserve the bill's validity in the face of flawed ministerial instructions, the drafter now deployed this symbol differently, shifting to prohibit ‘prohibited ingredients’. In so doing, prohibition was shorn from its usual statutory context, in which it symbolically sanctioned the use of operational regulatory devices, and instead was transformed into an explicit thing. Prohibition assumed a material form within cosmetics themselves, infusing their constituents with harmful capacities and unlawful status. Whereas previously the material ends of the bill were safe cosmetics, this drafting technique re-engineered those ‘ends as defined, limited, and even constituted by legal means’ (Riles, 2005: 1020). Cosmetics were being materialized, through law, as constituted of prohibited ingredients.

With this move, cosmetics were once again readied to be regulated. Yet the bill would not be tabled. Perhaps it collapsed under the weight of its internal contradictions, or perhaps Claxton lost his nerve. Whatever the exact reason, sometime in June 1946, the decision was made to strip all the cosmetics provisions from the bill. Claxton must have made this decision before the bill was submitted to Cabinet for approval on June 27, 1946. At that Cabinet meeting, ministers approved – without any changes – the proposed bill to amend the *Food and Drugs Act* (LAC, RG2, series A-5-a, Vol 2638, Items 7054 and 31906). When that bill was then introduced for first reading in the Senate on July 2, it made no mention of cosmetics (Bill X-9, 1946).

### Concluding thoughts

Faced with constitutional contestation around licensing, Canadian officials seriously considered registration as a mechanism to protect the public from harmful cosmetics. With its rejection in June 1946, another 60 years would pass before registration of cosmetics was required by Canadian law. In those six decades, National Health would lack the legal power to require industry to disclose information about the cosmetics that they made or sold in Canada.

The attempt to register cosmetics would seem to have come to naught, yet this controversy would have lasting effects. Rearranging relations between constitutional doctrine, legal form, regulatory devices, and injury, these events crystallized how cosmetics were conceived by Canadian officials. They highlight the midcentury turn, by federal drafters and solicitors, to the legal form of prohibition in health legislation. In *Proprietary Articles* in 1931, the Privy Council had made federal jurisdiction over criminal law a matter of form *over* substance; as translated by officials in 1946, prohibition was morphing into a form *for* substances. Moreover, these events solidified the notion that federal intervention on cosmetics – or on other confounding substances that the federal government wished to regulate – required the production of injury. Indeed, this story shows how injurious substances were both a precondition to, and produced by, federal cosmetics law. While injury was the legislative lynchpin, the injury brewed by the draft bill, in its relations between prohibition and registration, was neither static nor singular. Rather, cosmetic injury had been materially and temporally multiple, contoured by the very regulatory devices through which federal regulators would apprehend and grapple with these substances.

## Notes

1. Renamed in 1982, in Canada, as the *Constitution Act, 1867.*

2. Library and Archives Canada's database entry for this file is located at http://central.bac-lac.gc.ca/.redirect?app=fonandcol&id=3813552&lang=eng. This entry is out of date. In the conditions of access found under the sub-heading “Ordering and Viewing Options”, the entry incorrectly states that access to this file is “Restricted by law”. In fact, LAC released the file, in full, on January 29, 2018, after the author made a successful complaint to the Information Commissioner under the *Access to Information Act* (LAC file no. A201700062). The author maintains a copy on file.

3. This memorandum states “cold tar”, but Driedger surely meant “coal tar”.

4. In the US, the solution adopted for coal tar hair dyes was to cast them as adulterants *unless* the label bore a prescribed legend “warning the user of the risk of skin irritation and directing the user's attention to accompanying directions for preliminary skin tests” (Cavers, 1939: 41). See also US Food, Drug, and Cosmetic Act of 1938, §601(a).

5. Driedger cited *Rex v Warne Drug Co. Ltd*., [1917] 37 DLR 788 (ON SC), *Rex v Sheridan*, [1924] 3 DLR 339 (BC SC), and *Rex v Wakabayashi*, [1928] 1 WWR 487 (BC SC) (LAC, Driedger to Varcoe, 25 January 1946, Cosmetics File).

6. For accuracy, I note that *The Proprietary or Patent Medicine Act* excluded from this registration requirement drugs made to pharmacopoeial or regulated standards, and drugs that listed their ingredients on the label. That said, as of 1946, a small group of proprietary medicines under *The Proprietary or Patent Medicine Act* required both label declarations *and* registration. For ‘scheduled drugs’ – the 34 potent drug ingredients on the Schedule to the Act – it was mandatory to disclose such ingredients on a proprietary medicine's label (Soucy, 1953: 707).

7. For clarity, I do not have any complete drafts of the 1946 bill. In January 1946, Driedger opened his file on the constitutionality of the cosmetic licensing section, and later he placed on this file memoranda and correspondence regarding the 1946 bill (many of which included draft statutory text). However, he did not add his drafts of the 1946 bill to this file, but instead transmitted all completed drafts to National Health. Any file that National Health officials, or its legal advisor, may have maintained containing drafts of this bill appears not to have been archived.
